# Mortality in patients with Dupuytren’s disease in the first 5 years after diagnosis: a population-based survival analysis

**DOI:** 10.1177/17531934241235546

**Published:** 2024-03-15

**Authors:** Bente A. van den Berge, Feikje Groenhof, Paul M. N. Werker, Dominic Furniss, Rachel Kuo, Edwin R. van den Heuvel, Dieuwke C. Broekstra

**Affiliations:** 1 University of Groningen, University Medical Center Groningen, Department of Plastic Surgery, Groningen, the Netherlands; 2 University of Groningen, University Medical Center Groningen, Department of General Practice and Elderly Care Medicine, Academic General Practitioner Development Network, Groningen, the Netherlands; 3 University of Oxford, Nuffield Department of Orthopaedics, Rheumatology and Musculoskeletal Sciences, University of Oxford, Botnar Research Centre, Oxford, UK; 4 University of Oxford, Department of Plastic and Reconstructive Surgery, Oxford University Hospitals NHS Foundation Trust, John Radcliffe Hospital, Oxford, UK; 5 Eindhoven University of Technology, Department of Mathematics and Computer Science, Eindhoven, the Netherlands

**Keywords:** Dupuytren’s disease, mortality, survival, propensity score, epidemiology

## Abstract

Previous studies suggest that Dupuytren’s disease is associated with increased mortality, but most studies failed to account for important confounders. In this population-based cohort study, general practitioners’ (GP) data were linked to Statistics Netherlands to register all-cause and disease-specific mortality. Patients with Dupuytren’s disease were identified using the corresponding diagnosis code and assessing free-text fields from GP consultations. Multiple imputations were performed to estimate missing values of covariates, followed by 1:7 propensity score matching to balance cases with controls on confounding factors. A frailty proportional hazard model was used to compare mortality between both groups. Out of 209,966 individuals, 2561 patients with Dupuytren’s disease were identified and matched to at least four controls. After a median follow-up of 5 years, mortality was found to be actually reduced in patients with Dupuytren’s disease. There was no difference in mortality secondary to cancer or cardiovascular disease. Future studies with longer average follow-up using longitudinal data should clarify these associations in the longer term.

**Level of evidence:** III

## Introduction

Dupuytren’s disease affects 0.6%–31.6% of the population, most often men aged 50 years and older (Lanting et al., 2014). Previous studies have shown that multiple risk factors play a role in the development of Dupuytren’s disease, including advanced age, male sex, diabetes, alcohol consumption, smoking, manual work, low body mass index (BMI) and hyperlipidaemia, all of which are moderately to strongly associated with Dupuytren’s disease (Alser et al., 2020). In addition to these non-genetic and mostly modifiable risk factors, genetics also play a role in disease development. In fact, twin studies revealed that 80% of the disease heritability can be explained by genetic factors (Larsen et al., 2015), and two genome-wide association studies identified 26 loci that are involved in Dupuytren’s disease (Dolmans et al., 2011; Ng et al., 2017). Some of the pathways associated with these loci are also involved in the development of cancer (Dolmans et al., 2011).

With these findings, the long-standing view that Dupuytren’s disease is a local disease of the hands is shifting to the view that it is a systemic disease (Rehman et al., 2011). This idea is confirmed by studies reporting that having Dupuytren’s disease is associated with earlier death (Gudmundsson et al., 2002; Kuo et al., 2020; Mikkelsen et al., 1999; Wilbrand et al., 2005). In previous Scandinavian cohort studies, increased mortality was observed among patients with Dupuytren’s disease, although most of these studies did not control for any confounders for Dupuytren’s disease and mortality (Mikkelsen et al., 1999; Wilbrand et al., 2005). A recent study using a large population-based cohort comprising general practitioners’ (GP) data showed that having Dupuytren’s disease confers a survival benefit between 1 and 12 years after diagnosis but is associated with increased all-cause mortality beyond 12 years after diagnosis (Kuo et al., 2020). This increased mortality can be secondary to cancer, cardiovascular disease, respiratory disease, endocrine disease, digestive disease and psychiatric disease, including suicide and self-harm. Adjusting for smoking and diabetes reduced the effect size of increased mortality, which suggests that these factors are at least partly responsible for the increased mortality (Kuo et al., 2020). In this study, however, the analyses were not controlled for alcohol consumption, which is known to be a risk factor for both Dupuytren’s disease and mortality secondary to cancer or cardiovascular disease (Descatha et al., 2014; Kunzmann et al., 2018).

Before further research can be performed to investigate the causality of the excess mortality, the association between Dupuytren’s disease and mortality should first be further explored in another population-based cohort. Hence, the aim of this study was to compare the mortality rate in patients with Dupuytren’s disease and matched controls, using a large cohort of primary care date, accounting for important confounders.

## Methods

### Study design, data sources and population

In this population-based cohort study, data from the Academic General Practitioner Development Network (Academisch Huisarts Ontwikkel Netwerk [AHON]) were used. At the time of data extraction, this database comprised anonymized routinely collected care data of >358,000 patients from 50 affiliated registration practices located in the north of the Netherlands. The first data collection started in 1998. All data registered at the affiliated general practices are stored in the AHON database, including presenting complaints, symptoms and diagnoses, encoded using the International Classification of Primary Care (ICPC). The AHON data were linked to non-public data of Statistics Netherlands (Centraal Bureau voor de Statistiek or CBS), which collects and provides reliable statistical information and data, including national disease-specific mortality rates (CBS–Statistics Netherlands, n.d.). No medical ethical review was required according to the Dutch Medical Research Involving Human Subjects Act, because only registration data were used. Our study was conducted under AHON study ID 40. More details about the AHON cohort can be found at https://huisartsgeneeskunde-umcg.nl/ahon-database.

### Outcomes and data handling

All individuals included in the AHON database aged 40 years or older at the time of data extraction (31 March 2020) were selected, as Dupuytren’s disease rarely occurs in individuals aged <40 years. The primary outcome measure was all-cause mortality, and the secondary outcome measure was disease-specific mortality, classified according to the International Classification of Diseases (ICD-10) classification (Supplementary Table 1). Participants with Dupuytren’s disease were identified by selecting individuals who had the ICPC code for Dupuytren’s disease (L99.03). In addition, all consultation notes documented in free-text fields were searched. All notes containing the word ‘Dupuytren’, ‘koetsiersziekte’ or ‘koetsiershand’ – disease names in common parlance – were assessed to justify the diagnosis. Other ways of spelling were checked in a subsample, but this did not yield a significant addition. If there was no Dupuytren’s disease present according to the GP notes or when the notes were inconclusive and no diagnosis code for Dupuytren’s disease was registered, individuals were labelled as controls. For all patients with Dupuytren’s disease, the date of diagnosis was registered by selecting the date of first registration of the diagnosis code or ‘Dupuytren’ etc. in the free-text fields.

### Confounders

Of all individuals, the presence of the following potential confounders was assessed by using the corresponding ICPC codes: diabetes; excessive smoking; alcohol abuse; overweight; and fat metabolism disorders (Supplementary Table 2). To determine which individuals had died and identify the cause of death, our dataset was linked to the CBS database by using a pseudonym based on sex, date of birth and postal code. For the AHON database, this pseudonym was generated by ZorgTTP as the trusted third party (TTP). Individuals might be included in the AHON dataset more than once, most likely due to moving to another AHON-registered general practice. In that case, the duplicate records were merged into one record. After data linkage, the date and cause of death of all deceased individuals were registered. In addition, we registered the household income and highest level of education reached, because both the socioeconomic status and level of education are related to self-reported health and lifestyle factors, such as smoking, physical exercises and diet (Alkerwi et al., 2015; Beenackers et al., 2012; Casetta et al., 2017). Therefore, these variables were used as proxy variables for lifestyle factors. Level of education was ranked according to the International Standard Classification of Education (ISCED) 2011 of UNESCO (United Nations Educational Scientific and Cultural Organization (UNESCO), 2011) (Supplementary Table 3). The household income percentile of each individual has been registered for each calendar year. The earlier the variable is registered, the better it is as a predictor for developing Dupuytren’s disease and mortality. Therefore, the first registration year of this variable was used, which was 2011.

### Statistical analysis

Baseline characteristics were reported by means and standard deviations (SDs) for normally distributed continuous variables and by medians and interquartile ranges (IQRs) for non-normally distributed continuous variables. For dichotomous variables, frequencies and proportions were reported.

*Multiple imputation* – The variables household income and level of education contained 7% and 52% missing values, respectively. The CBS has collected educational data from various registers and the Occupational Population Survey (EBB) since 1999. This database does not cover the complete Dutch population yet, which explains the high proportion of missing values. Multiple imputation with chained equations with the R package *mice* was performed (van Buuren and Groothuis-Oudshoorn, 2011) by predictive mean matching using date of birth, sex, diabetes, excessive smoking, alcohol abuse, overweight and fat metabolism disorders, Dupuytren’s disease, the median household income for the corresponding postal code (registration year 2015) and the household income of 2012 up to and including 2020. Six imputed datasets with 20 iterations were created (Rubin, 1987).

*Propensity score matching* – Propensity score matching (PSM) was applied using the R package *MatchThem* (Pishgar et al., 2021) to balance cases and controls on all covariates, in all six imputed datasets. All cases of Dupuytren’s disease were matched with unaffected controls on propensity score. Propensity scores were calculated by logistic regression modelling with Dupuytren’s disease as the outcome and with potential confounders and factors related to the outcome (Brookhart et al., 2006): age; sex; diabetes; excessive smoking; alcohol abuse; overweight; and fat metabolism disorders. Age was included as a continuous variable and the others as binary variables. Including income and level of education in the PSM worsened the overall balance. Therefore, it was decided to run the analysis twice: both adjusted and unadjusted for income and level of education. The nearest neighbour method without replacement was applied with a calliper width at 0.2 SD of the logit propensity score. A 1:7 matching of cases to controls was applied. The balance of covariates distribution between cases and controls was assessed with the standardized mean differences (SMD) and balance plots (Rubin, 2004).

*Survival analysis* – A frailty proportional hazards model was used using the R package *survival* (Therneau, 2022), with time to death or censoring during the follow-up period as the outcome. The frailty is a random effect for the hazard rate to allow for heterogeneity in hazard rates across matched individuals. For cases, the follow-up started at the date that Dupuytren’s was diagnosed (i.e. the index date). For their matched controls, the same index date was selected. The end of the follow-up period was determined as the date of data retrieval (31 March 2020) or the date of deregistration from the general practice or death, whichever came first. Controls who died before their index date or were deregistered from their general practice before their index date were excluded. By using a relatively high matching ratio of 1:7, fewer cases were lost because most cases had at least one matched control left. The analyses were performed both unadjusted and adjusted for income percentile (continuous) and level of education (ordinal variable). The analyses were repeated for each imputed dataset and the results were pooled using Rubin’s rules. Results were presented as hazard ratios (HR) with 95% confidence intervals (CIs). For each ICD-10 cause of death category with sufficient events (>20), a disease-specific analysis was performed using the chi-square test. A *p*-value <0.05 was considered significant.

*Simulation* – In theory, excluding controls who died or were deregistered from their general practice before their index date may introduce bias, as this could have led to a lower number of individuals at risk of death in the control group. This potential bias was assessed by performing a simulation study with 290,000 individuals and repeating it 1000 times. The results showed that there was low risk of bias. The methods and results of this simulation are described in Supplementary Methods 1.

## Results

### Selection of study population

**Figure 1.**
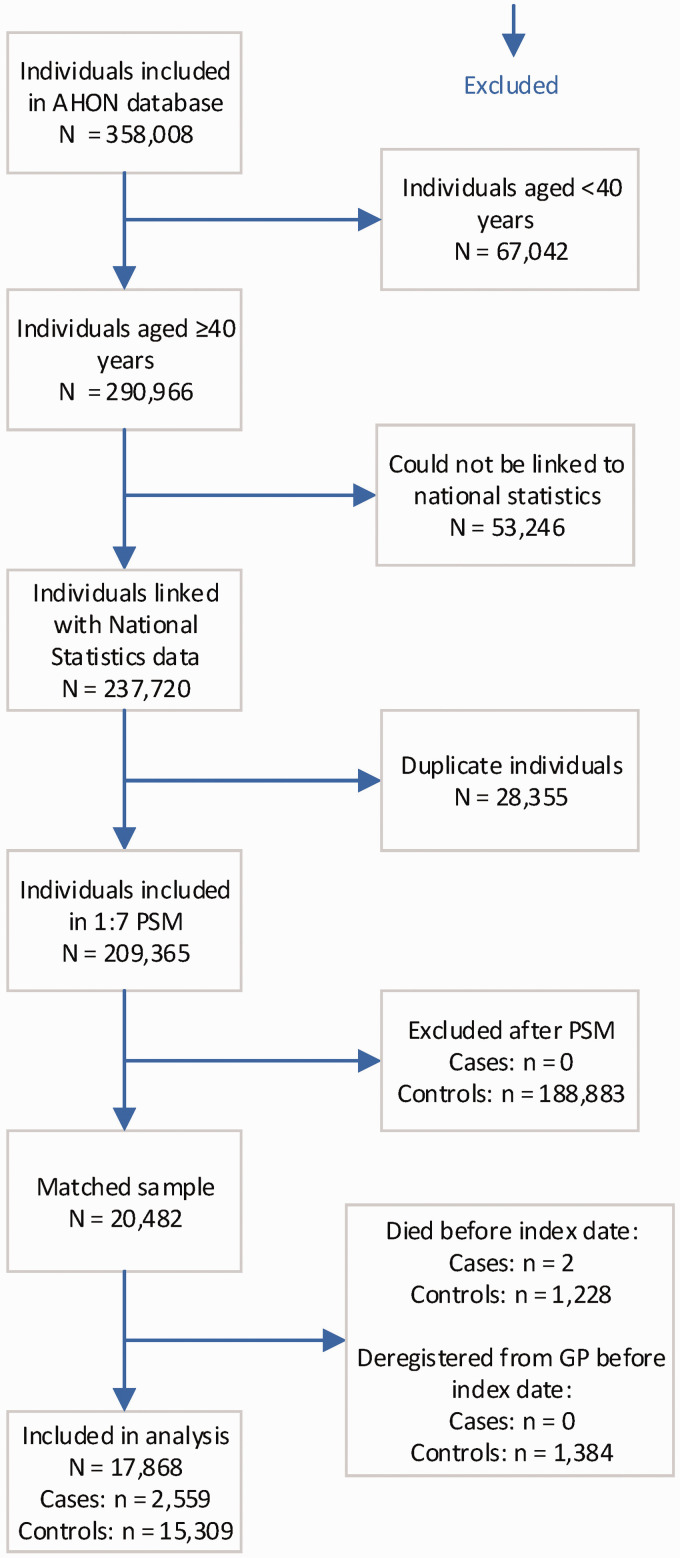
Flow chart of the patient inclusion process.

Out of 290,966 individuals aged ≥40 years, 2870 (1.0%) patients with Dupuytren’s disease were identified in the AHON data, based on the ICPC code for Dupuytren’s disease and GP notes. In total, 141 individuals were assigned to the control group because there was no Dupuytren’s disease present according to the GP notes, and 115 individuals because the GP notes were inconclusive. The remaining 2614 patients were identified as patients with Dupuytren’s disease. Out of the total cohort, 237,720 (81.7%) could be linked to the CBS data. Only 37 patients with Dupuytren’s disease (1.4%) could not be linked (Supplementary Table 4). In total, 28,355 individuals, of which 16 were patients with Dupuytren’s, appeared to be included more than once in the AHON dataset. Therefore, 209,365 unique individuals were included in the PSM. All but two cases could be matched to seven controls. The remaining two cases could be matched to four controls. Out of 20,482 matched cases and controls, 1228 (6.0%) controls died and 1384 (6.8%) controls deregistered before the index date of their case. Furthermore, two cases died before their index date, most likely due to an input error of the date of diagnosis by the GP. Hence, 17,868 (87.2%) individuals were included in the analysis (Figure 1).

### Baseline characteristics

Before matching, cases were older and more often male than controls, and cases were more often diagnosed with diabetes and fat metabolism disorders (Table 1). After matching, both groups were balanced, as the SMDs of all covariates were reduced to <0.1. The median age at diagnosis of Dupuytren’s disease (i.e. index date) was 63 years (IQR 55–70). Most cases (*n* = 1583, 61.9%) and matched controls (*n* = 9549, 61.8%) were men (Table 1).

**Table 1.** Descriptive statistics of the study population, before and after PSM.

	Unmatched			Matched		
	Cases	Controls	SMD	Cases	Controls	SMD
Number	2561	206,804		2559	15,309	
Age (years)^a^	69 (62–76)	62 (51–75)	0.391	69 (62–76)	69 (62–76)	0.024
Age at index date (years)	NA	NA	NA	63 (55–70)	63 (55–69)	0.060
Sex (M)	1585 (61.9)	101,121 (48.9)	0.268	1583 (61.9)	9459 (61.8)	0.002
DM	557 (21.7)	24,348 (11.8)	0.242	556 (21.7)	3444 (22.5)	−0.019
Excessive smoking	212 (8.3)	16,763 (8.1)	0.006	211 (8.2)	1301 (8.5)	−0.009
Alcohol abuse	71 (2.8)	3172 (1.5)	0.077	71 (2.8)	306 (2.0)	0.051
Overweight	147 (5.7)	12,675 (6.1)	−0.017	147 (5.7)	867 (5.7)	0.003
Fat metabolism disorder	559 (21.8)	25,877 (12.5)	0.226	557 (21.8)	3701 (24.2)	−0.057
Income (percentile)^b^	55.8 (SD 26.1)	51.6 (SD 26.4)	0.160	55.8 (SD 26.1)	53.6 (SD 26.3)	0.084
Education (ISCED level)^b^			0.048			0.069
Early childhood (0) or primary (1)	394 (15.4)	32,129 (15.5)		394 (15.4)	2726 (17.8)	
Lower secondary (2)	539 (21.0)	40,032 (19.4)		537 (21.0)	3278 (21.4)	
Higher secondary (3)	992 (38.7)	81,296 (39.3)		992 (38.8)	5968 (39.0)	
Post-secondary non-tertiary (4) or short cycle tertiary (5)	78 (3.0)	5804 (2.8)		78 (3.0)	426 (2.8)	
Bachelor (6), Master (7) or PhD (8)	559 (21.8)	47,514 (23.0)		559 (21.8)	2910 (19.0)	

Data expressed as *n* (%), mean (SD) or median (IQR).

^a^Expected age of participants at date of extraction (31 March 2020) when all participants would be alive.

^b^Mean and SD (income) and frequencies and proportions (level of education) reported are the pooled values of the six imputed datasets. Standard error of the mean of income = 0.03 (Rubin’s rules). Pooled mean frequencies are rounded to the nearest integer.

DM: diabetes mellitus; IQR: interquartile range; ISCED: International Standard Classification of Education; PSM: propensity score matching; SD: standard deviation; SMD: standardized mean difference.

### Mortality

**Figure 2.**
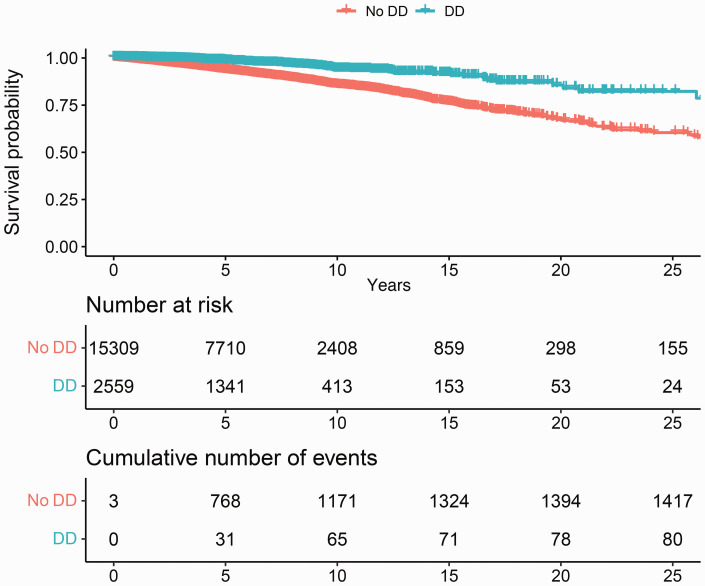
Kaplan–Meier curve of the survival probability showing a decreased mortality of patients with Dupuytren’s disease compared to matched controls.

The median follow-up from index date until death or censoring was 5.1 years (IQR 2.5–8.3). In total, 1538 (8.6%) out of 17,868 individuals had died during our follow-up period. Out of 2559 cases, 82 (3.2%) had died, compared to 1456 (9.5%) out of 15,309 controls. The mean age of death in patients with Dupuytren’s was 79.5 years (SD 9.8), and 76.2 years (SD 9.5) in matched controls. Patients with Dupuytren’s disease were at lower risk of death than matched individuals without Dupuytren’s disease (unadjusted pooled HR 0.24, 95% CI: 0.20 to 0.30). The results were almost the same after adjustment for income and level of education (adjusted pooled HR 0.25, 95% CI: 0.20 to 0.31, *p* <0.001) (Figure 2, Table 2, Supplementary Table 5).

**Table 2.** Results of the chi-square test of the main causes of death in our study population, classified with the ICD-10 classification.

Cause of death	Cases	Controls	Total	Chi-square test value	*p*
All-cause	82	1456	1538	NA	NA
Cancer	35 (43)	546 (38)	581 (38)	0.450	0.502
Cardiovascular disease	23 (28)	381 (26)	404 (26)	0.142	0.706
Other disease^a^	24 (29)	529 (36)	553 (40)	1.682	0.195

Results based on calculations using non-public microdata from Statistics Netherlands. Data expressed as *n* (%).

a ‘Other disease’ comprises death causes from 14 different ICD-10 categories. The numbers per category were too small to be analysed separately.

### Disease-specific mortality

The most common cause of death was cancer (*n* = 581, 38%), followed by cardiovascular disease (*n* = 404, 26%). The proportion of Dupuytren’s disease that died from cancer (35 of 82 deaths) did not differ from the proportion of controls (581 of 1456 deaths) that died from cancer (chi-square = 0.450, *p* = 0.502) and cardiovascular disease (chi-square = 0.142, *p* = 0.706). The events in other ICD-10 categories in the Dupuytren’s group were too small to be analysed separately.

## Discussion

In this population-based cohort study, we conducted a propensity score matched analysis to compare mortality between patients with Dupuytren’s disease and controls. Our results showed that patients with Dupuytren’s disease have a lower mortality risk than matched controls in the first years after diagnosis.

Mortality in patients with Dupuytren’s disease has been the subject of several previous studies (Gudmundsson et al., 2002; Kuo et al., 2020; Mikkelsen et al., 1999; Wilbrand et al., 2005). All these studies had concluded that Dupuytren’s disease was associated with increased mortality. At first sight, their results seem contradictory with our findings that Dupuytren’s disease is associated with reduced mortality. However, after examining the results of these studies in more detail, some similarities were found with our study. In a large study among surgically treated patients (Wilbrand et al., 2005), reduced mortality was observed in the total population within the first 4 years after diagnosis and in a subgroup of patients aged ≥70 years at the time of surgical treatment during the complete follow-up of ≥15 years. In our study population, the median age at diagnosis in primary care was 63 years. Given that several years had elapsed between diagnosis and first treatment (Broekstra et al., 2023), it is conceivable that most included patients would have been treated at the age of ≥70 years, and that our study population is comparable with this subgroup. In another study among 1287 male patients (Gudmundsson et al., 2002), an increased mortality was found in patients with (treated) joint contractures during 15 years of follow-up, but not in patients with only palpable nodules. The results of these previous studies suggest that patients with a high genetic risk for Dupuytren’s disease, corresponding with an early age of onset and more advanced disease (Dolmans et al., 2012), are at risk of increased mortality, while patients with mild disease and later age of onset are not. In our study, the stage of Dupuytren’s disease was unknown. Therefore, our patients could not be stratified on disease severity to further investigate this hypothesis. In another study using comparable methodology, i.e. GP data but with a much larger dataset (Kuo et al., 2020), a reduced mortality was found in the first 12 years after diagnosis, but which increased from 12 years after diagnosis. The authors accounted for several confounders, but not for excessive alcohol consumption, which is a known risk factor for both Dupuytren’s disease and mortality (Descatha et al., 2014; Kunzmann et al., 2018). The ‘flip’ in mortality was also observed in another study (Mikkelsen et al., 1999), in which patients aged 61–69 years at screening for Dupuytren’s disease seem to have a reduced mortality only in the first 11 years approximately. In this study, however, the authors did not control for any confounders. Therefore, it is unclear whether the flip would have remained if these studies would have controlled for all known confounders and factors related to the outcome. Potentially, the follow-up in our study was too short to observe a flip from reduced to increased mortality after a certain number of years.

The majority of included individuals died of cancer (38%) or cardiovascular disease (26%). Our results showed that the number of patients that died of cancer or cardiovascular disease did not differ significantly between cases and controls. These results are not in line with previous studies with longer follow-up and larger sample sizes, reporting that patients with Dupuytren’s disease die more often from cancer and cardiovascular disease. For some causes of death, such as cardiovascular disease, increased mortality was only observed 10–12 years after diagnosis, while in the first years after diagnosis or surgery, mortality of cardiovascular disease was reduced in patients with Dupuytren’s disease (Kuo et al., 2020; Wilbrand et al., 2005).

Most previous studies did not control for the confounding effect of lifestyle factors – or socioeconomic factors, which can be regarded as proxy for lifestyle factors – such as smoking, alcohol consumption and co-morbidities such as diabetes mellitus (DM), obesity and fat metabolism disorders (Gudmundsson et al., 2002; Mikkelsen et al., 1999; Wilbrand et al., 2005). This could have affected the results of all-cause and disease-specific mortality. The study by Kuo et al. (2020) included smoking, BMI and DM in their final model, but did not include alcohol consumption. Their results showed that adjusting for smoking and DM reduced the effect estimates of increased mortality, suggesting that these factors are at least partially responsible. Our results suggest that having Dupuytren’s disease protects from an earlier death when accounting for even more confounders, such as excessive alcohol intake, at least in the first years after diagnosis. It remains unclear whether patients with Dupuytren’s disease are at reduced or increased risk of cancer or cardiovascular disease or other diseases on the longer term.

Our study has several strengths. First, GP data were used, resulting in the inclusion of patients diagnosed in primary care, which enhances the generalizability of our findings compared to other studies that included only surgically treated patients. By identifying patients using both diagnosis codes and assessing GP notes, the chance of misclassification was reduced. Furthermore, PSM was used to balance cases and controls. PSM is preferred over regression adjustment to remove bias if the covariate distribution differs widely between cases and controls, which was the case in our study (Baser, 2007). Lastly, by using income and level of education as proxy variables for lifestyle factors, and including an alcohol variable, our study adds to the current literature, and is an external validation of established work in a different country and dataset.

The present study has some limitations. First, after linking our data to the Statistics Netherlands registry, a considerable number of individuals (18%) could not be linked. Possible reasons are that individuals moved to a non-affiliated practice without deregistering from their previous practice, a change in the software system of an affiliated general practice or errors in the postal code or date of birth of individuals. Our linkage percentage is in line with the expected linkage percentage of 80%–90% when using the pseudonym linkage based on sex, date of birth and postal code (National Statistics (CBS), 2022). However, unlinked individuals were approximately 5 years older than individuals who could be linked (Supplementary Table 4). Hence, bias might have been introduced if this inability to link did not occur randomly. Nevertheless, all our cases of Dupuytren’s disease could be matched with at least one control with the same propensity score because the size of our study population was sufficient. Second, diagnosis codes were used to identify confounders. The number and specificity of codes for smoking, alcohol consumption and obesity are, however, limited, and the registration of these codes depends on the registration behaviour and judgement of GPs. For example, GPs can only register a code for ‘alcohol abuse’. It is likely that GPs do not assess excess alcohol consumption or smoking or obesity when there is no possible related health issue. Therefore, these variables are likely subjected to bias. In addition, smoking, alcohol consumption and obesity could not be quantified, although studies have shown that the risk on Dupuytren’s disease increases with increasing alcohol consumption and lower BMI (Godtfredsen et al., 2004; Hacquebord et al., 2017). For this reason, our analysis was controlled for income and level of education, as both factors are known to be related to smoking, alcohol consumption and body weight and can therefore be considered proxy variables (Casetta et al., 2017; Cerdá et al., 2011; Kim, 2016; Schnohr et al., 2004). Lastly, in our study, the prevalence of Dupuytren’s disease was 1.2%, while the true prevalence in the general population is much higher (Lanting et al., 2013). This suggests that most patients with Dupuytren’s disease do not seek medical care for their symptoms. Accordingly, our population could represent patients with a higher health-seeking behaviour, who may be more willing to modify risk factors or receiving timely treatment for life-threatening diseases. It was impossible to account for this factor, for example by matching for number of GP appointments in the 12 months before diagnosis (Clewley et al., 2018), in our analysis.

In conclusion, this study showed that a reduced overall mortality was observed in patients diagnosed with Dupuytren’s disease during the first 5 years after diagnosis. Future observational studies with longitudinally collected healthcare and lifestyle date with a long follow-up should clarify the association between Dupuytren’s disease and mortality in the longer term.

## Supplemental Material

sj-pdf-1-jhs-10.1177_17531934241235546 - Supplemental material for Mortality in patients with Dupuytren’s disease in the first 5 years after diagnosis: a population-based survival analysis Supplemental material, sj-pdf-1-jhs-10.1177_17531934241235546 for Mortality in patients with Dupuytren’s disease in the first 5 years after diagnosis: a population-based survival analysis by Bente A. van den Berge, Feikje Groenhof, Paul M. N. Werker, Dominic Furniss, Rachel Kuo, Edwin R. van den Heuvel and Dieuwke C. Broekstra in Journal of Hand Surgery (European Volume)
