# Supplementary material for: Mortality in patients with Dupuytren’s disease in the first 5 years after diagnosis: a population-based survival analysis
Source: J Hand Surg Eur Vol. 2024 Mar 15;49(9):1110–8. doi: 10.1177/17531934241235546 (PMC11457468; doi:10.1177/17531934241235546)
Supplement: sj-pdf-1-jhs-10.1177_17531934241235546 - Supplemental material for Mortality in patients with Dupuytren’s disease in the first 5 years after diagnosis: a population-based survival analysis [file sj-pdf-1-jhs-10.1177_17531934241235546.pdf]

SUPPLEMENTARY MATERIAL

Supplementary Table 1: International classification of disease (ICD-10).

Category	Description
1	Certain infectious and parasitic diseases
2	Neoplasms
3	Diseases of the blood and blood-forming organs and certain disorders involving the immune mechanism
4	Endocrine, nutritional and metabolic diseases
5	Mental, Behavioral and Neurodevelopmental disorders
6	Diseases of the nervous system
7	Diseases of the eye and adnexa
8	Diseases of the ear and mastoid process
9	Diseases of the circulatory system
10	Diseases of the respiratory system
11	Diseases of the digestive system
12	Diseases of the skin and subcutaneous tissue
13	Diseases of the musculoskeletal system and connective tissue
14	Diseases of the genitourinary system
15	Pregnancy, childbirth and the puerperium
16	Certain conditions originating in the perinatal period
17	Congenital malformations, deformations and chromosomal abnormalities
18	Symptoms, signs and abnormal clinical and laboratory findings, not elsewhere classified
19	Injury, poisoning and certain other consequences of external causes
20	External causes of morbidity
21	Factors influencing health status and contact with health services

Supplementary Table 2: Diagnosis codes used to identify Dupuytren's disease patients and risk factors

**Diagnosis**	**ICPC-code used:**	**Meaning**
Dupuytren's disease	L99.03	'Dupuytren's contracture'
Diabetes	T90 or T90.01 or T90.02	'Diabetes Mellitus' or 'diabetes mellitus type 1' or 'diabetes mellitus type 2'
Excessive smoking	P17	'Tobacco abuse'
Alcohol abuse	P15 or P15.01 or P15.02 or P15.05 or P15.06	'Chronic alcohol abuse' of 'alcoholism' or 'delirium tremens' or 'problematic alcohol use' or 'Binge drinking'
Fat metabolism disorder	T93 or T93.01 or T93.02 or T93.03 or T93.04	'Fat metabolism disorder' or 'hypercholesterolemia' or 'hypertriglyceridemia' or 'mixed hyperlipidemia' or 'familial hypercholesterolemia/hyperlipidemia'
Overweight	T82 or T83	'Adiposity' or 'overweight'

Supplementary Table 3: International Standard Classification of Education (ISCED) 2011 to determine the highest education level reached

**ISCED level**	**Description**
Level 0	Early childhood education
Level 1	Primary education
Level 2	Lower secondary education
Level 3	Upper secondary education
Level 4	Post-secondary non tertiary education
Level 5	Short cycle tertiary education
Level 6	Bachelor's or equivalent level
Level 7	Master's or equivalent level
Level 8	Doctoral or equivalent level

Supplementary Table 4: Characteristics of individuals that could be linked to National statistics, and individuals that could not be linked.

	Not linked to National Statistic	Linked to National Statistics	Total
N (%)	53,246 (18)	237,720 (82)	290,966 (100)
Age*, median (IQR)	67 (51 – 92)	62 [51 – 75]	62 [51 – 77]
Sex, m (%)	27,329 (51.3)	116,389 (49.0)	143,718 (49.4)
DM (%)	2,692 (5.1)	25,297 (10.6)	27,989 (9.6)
Excessive smoking (%)	727 (1.4)	17,245 (7.3)	17,972 (6.2)
Alcohol abuse (%)	299 (0.6)	3,317 (1,4)	3,616 (1.2)
Overweight (%)	649 (1.2)	13,041 (5.5)	13,690 (4.7)
Fat metabolism disorder (%)	1,370 (2.6)	26,835 (11.3)	28,205 (9.7)
Dupuytren's disease (%)	37 (0.1)	2,577 (1.1)	2,614 (0.9)

* Expected age of participants at date of extraction (31-3-2020) when all participants would be alive

Supplementary Table 5: Results of the Cox regression analysis, presented for each imputed dataset separately

All-cause mortality	HR (95% CI)	p
**Dataset 1**		
DD	0.248 [0.199 – 0.309]	**<0.001**
Education level	0.969 [0.811 – 1.157]	0.726
Income	0.994 [0.991 – 0.996]	**<0.001**
**Dataset 2**		
DD	0.249 [0.200 – 0.310]	**<0.001**
Education level	1.035 [0.871 - 1.230]	0.694
Income	0.993 [0.990 – 0.995]	<0.001
**Dataset 3**		
DD	0.249 [0.200 – 0.310]	**<0.001**
Education level	0.895 [0.751 – 1.067]	0.216
Income	0.993 [0.991 – 0.996]	**<0.001**
**Dataset 4**		
DD	0.248 [0.200 – 0.309]	**<0.001**
Education level	0.928 [0.772 – 0.116]	0.426
Income	0.994 [0.991 – 0.995]	**<0.001**
**Dataset 5**		
DD	0.250 [0.201 – 0.311]	**<0.001**
Education level	0.912 [0.766 – 1.088]	0.307
Income	0.994 [0.992 – 0.997]	**<0.001**
**Dataset 6**		
DD	0.251 [0.201 – 0.312]	**<0.001**
Education level	0.752 [0.628 – 0.899]	**0.002**
Income	0.994 [0.992 – 0.996]	**<0.001**
**Pooled analysis**		
DD	0.249 [0.200 – 0.310]	**<0.001**
Education level	0.911 [0.662 – 1.253]	0.537
Income	0.994 [0.991 – 0.996]	**<0.001**

Supplementary Methods 1

The simulation study was intended to investigate the matching procedure where controls could be eliminated due to inappropriate ordering of events (e.g., death before the date of diagnosis). The goal was to study a possible bias in the estimation of the hazard ratio when cases and controls have the same survival distribution, i.e., no effect of Dupuytren disease (DD). If our matching procedure is unbiased, we should observe a log hazard ratio of zero and a probability of 0.05 for falsely rejecting the null hypothesis of no effect of DD. We simulated 290000 participants, approximately the number of participants we originally collected for the analysis, and evaluated their survival status at the year 2020. We used the underlying distributions of the data to mimic the age and survival distribution. The simulation and analysis were done in SAS software. We repeated this simulation 1000 times to determine the potential bias and the type 1 error rate.

Simulation of Data

We first simulated a birth year (B) for 290000 participants within the period 1920 to 1980. We restricted participants to a minimum age of 40 years (since we excluded younger people from the data) and a maximum age of 100 years. Thus, we simulated on average 4755 participants for each year.

Now that we know the year of birth, we need to simulate an age at death (T_D) for each participant. We used the Weibull distribution (with 2.48 as the shape parameter and 27.68 as the scale parameter) and added 40 years (since the Weibull starts at zero).

We used a Bernoulli random variable (Z) with probability 0.02 to generate participants with DD, leading to 5800 cases per simulation study on average. For these participants we generated an age (T_{DD}) at which they are diagnosed with DD using a normal distribution with mean 63 years and standard deviation of 10 years. We eliminated participants with an age of DD that was beyond the age of death or when the diagnosis would occur after the year 2020 (i.e., we removed cases with $T_{DD} > \min\{T_D, 2020 - B\}$). On average we removed 63.2% of the cases, resulting in 2133 cases per simulation study on average available for matching.

Since we did not simulate any covariates, we used the year of birth to match the controls for each case, i.e., using information that is unrelated to the diagnosis and survival. For each case we randomly selected 7 (without replacement) participants of the control group having the same birth year. Since we have ample participants per year of birth, we created on average 2133 matched sets of eight participants with one participant having DD and the others being controls.

Preparing Data for Analysis

To be able to analyze the matched sets of cases and controls, we need to (1) eliminate controls that died before the age of diagnosis of the matched case and (2) create survival data (i.e., a censoring variable C and a survival time S) for controls and cases.

Elimination of controls: Since the cases and controls have the exact same birth year, we can directly eliminate controls with $T_D^{\text{Control}} < T_{DD}$, where T_{DD} is the age of diagnosis of DD for the case in the matched set and T_D^{Control} is the age of death for a control in the matched set. On average we have removed 26.7% of the controls, resulting in 5.13 controls per case on average.

Creating survival data: If the death of a participant occurs after 2020 (i.e., $B + T_D > 2020$), the participant did not yet die in 2020 and the censoring variable for this participant becomes one ($C = 1$), otherwise the censoring variable is zero ($C = 0$). If the participant is censored, the age of the participant in 2020 is equal to $A = 2020 - B$. If the participant died before 2020, the age of the participant at death is $A = T_D$. This calculation of age would be true for both the cases and controls. In the survival analysis we used the follow-up time, counted from the age

of diagnosis of the case in the matched set. Thus, the survival time for the participants in a matched set is calculated by $S = A - T_{DD}$, with T_{DD} the age of diagnosis of DD for the case in the matched set.

Results of Analysis

Descriptive statistics for the age and follow-up times (over all 1000 simulations) are reported in Table 1 for the cases and controls separately. The overall censoring percentage was 30.5% and 29.5% for the controls and cases, respectively.

Table 1: Descriptive statistics of the age and follow-up for cases and controls in the simulation study.

	Controls			Cases		
	Median	Mean	Std.	Median	Mean	Std.
Age	64.9	65.1	8.93	66.2	66.4	8.90
Follow-up	9.57	11.4	8.65	8.63	10.5	8.29

It shows that there is a minimal difference in characteristics of the age and follow-up times for the (censored) event. Figure 1 shows the histograms of the age of the first simulation data set. Again, no real differences are demonstrated in the age distribution.

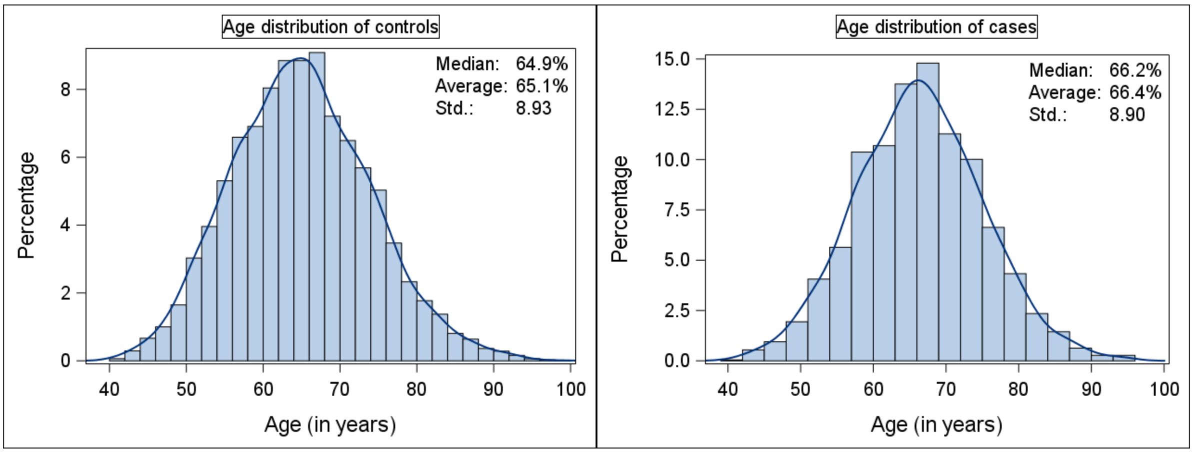

Figure 1: Age distribution of cases and controls for the first simulation data set.

Figure 2 shows the Kaplan-Meier curves for the cases and controls of the first simulation data set. Here we see a small difference in the survival curves between 5 to 20 years of follow-up times. The survival of controls is slightly better than for the cases (but the differences are relatively small).

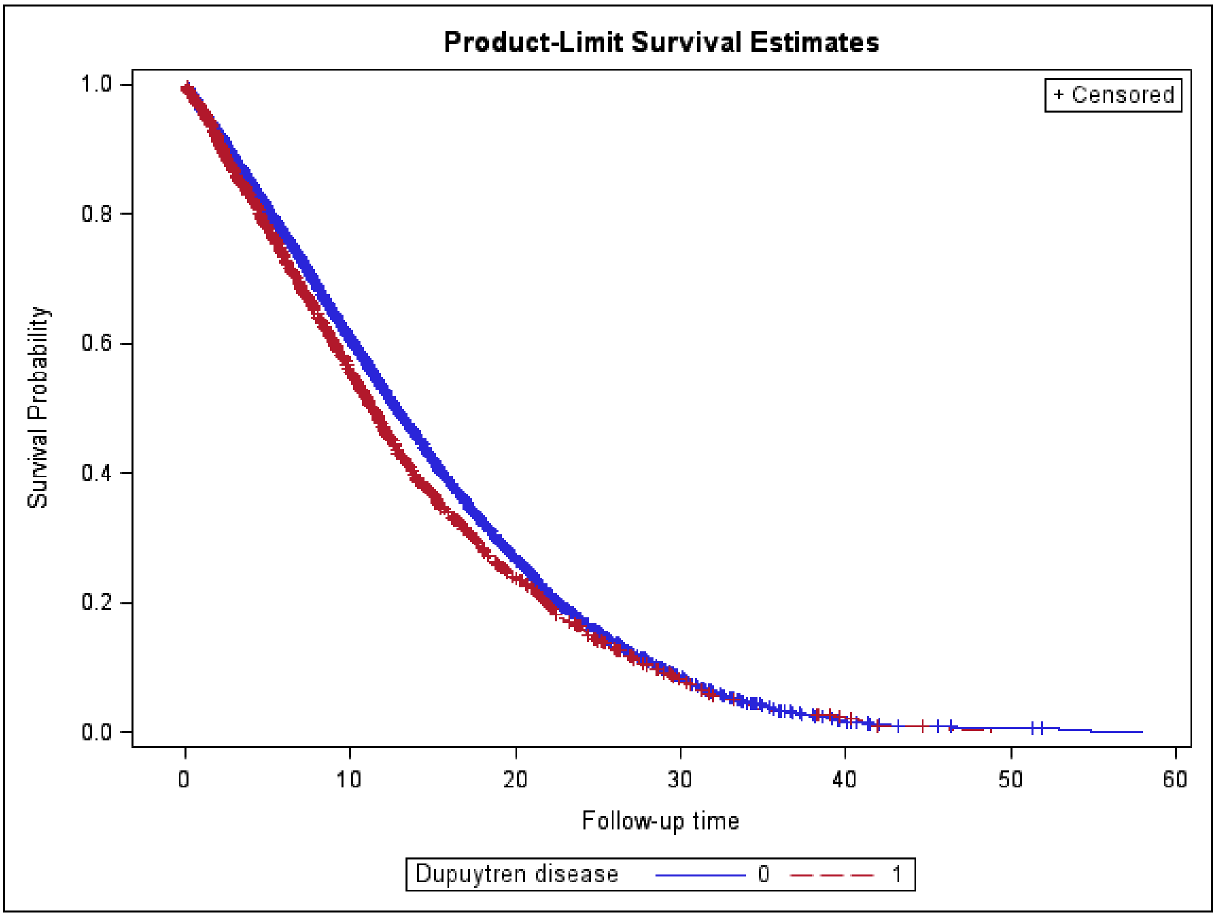

Figure 2: Kaplan-Meier curves for the cases and controls of the first simulation data set.

The Cox proportional hazard analysis (stratified for the matched sets of participants) of the follow-up times shows that the average log hazard ratio (over 1000 simulations) was estimated at 0.0006 with a standard error of 0.031. A value of zero would indicate a hazard ratio of one and no bias. The minimum log hazard ratio over the 1000 simulations was determined at -0.079 (HR: 0.924) and the maximum was determined at 0.109 (HR: 1.115). Finally, the type 1 error rate for rejecting the null hypothesis of "no effect" was determined at 3.8%, which is a little bit conservative. Thus, these simulation statistics supports that our matching procedure is unbiased when it comes to the estimation of the hazard ratio for DD, while the type 1 error rate indicates that we do not reject the null hypothesis too quickly.
